# Root-Filling

**Published:** 1904-09

**Authors:** T. Wilson Hogue

**Affiliations:** Vermont, Bournemouth, England


### ROOT-FILLING.[1]

BY T. WILSON HOGUE, D.M.D., VERMONT, BOURNEMOUTH, ENGLAND.

Mr. President and Gentlemen,—I have such an exalted opinion of American dentistry that it is with the greatest diffidence I lay this short paper before you.

It seems like sending coals to Newcastle to send a paper on dentistry to America. Nevertheless it may interest some of the younger members of the Harvard Dental Alumni Association to hear the experience of one who has tried not to work in a groove, but fairly to try all new methods of practice that at all promised success, and has done so during many years.

When the pulp of a tooth is exposed, whether by decay or an excavator, my practice is not to cap, but destroy. Capping has such an element of uncertainty about it that I favor extirpation of the pulp, and use S. S. White's devitalizing fibre, sometimes incorporating with it a minute quantity of S. S. White's nerve-paste, being most careful that the application is properly sealed in the cavity.

Excelsior, or Donaldson's pulp-canal cleansers, are in my hands the best instruments for removing the pulp, and I only use the finest size. In a large canal I insert two of the cleansers and sometimes, say in a large upper canine, three, rotating them together.

Mummifying pastes I have not found reliable, and I have given them up for a better and more certain treatment.

Free and easy access to the canals must be made, however much tissue has to be sacrificed, and very small canals sometimes slightly enlarged with suitable drills,—Gates-Glidden and Bentel-rock's are excellent.

My experience is that the apex of the root should not be perforated, and I am very careful to avoid perforation.

For disinfecting septic canals I use perchloride of mercury or oil of cloves.

Oxychloride of zinc I have found by far the best material for filling canals, as it remains sweet and pure indefinitely.

---

[1] Read before the Harvard Dental Alumni Association, "Alumni Day," June 27, 1904.

Stiffened paper points for drying pulp-canals I use extensively, and find useful.

Gutta-percha in every form has proved with me very inferior to oxychloride of zinc; if the former filling is removed from a root, it always smells nasty, although it may only have been there for a few weeks.

It is a great help to have a large assortment of suitably curved instruments for inserting dressings, as well as for permanently filling the canals of roots.

Although charcoal has only been used by me for a few months, yet in concluding this short paper I wish to say that I have found it an exceedingly valuable filling, and as it is practically insoluble it ought to prove a permanent one also.

This paper will, I hope, be the means of leading to an interesting discussion.

I do not know exactly how it is in America, but I believe that in England no operation is so often imperfectly performed as the filling of roots. It seems to me no operation in dentistry—I had almost said in surgery—demands more time, skill, and care.

